# Vitamin Status among Breastfed Infants in Bhaktapur, Nepal

**DOI:** 10.3390/nu8030149

**Published:** 2016-03-08

**Authors:** Manjeswori Ulak, Ram K. Chandyo, Andrew L. Thorne-Lyman, Sigrun Henjum, Per M. Ueland, Øivind Midttun, Prakash S. Shrestha, Wafaie W. Fawzi, Lauren Graybill, Tor A. Strand

**Affiliations:** 1 Department of Child Health, Institute of Medicine, Tribhuvan University, Maharajgunj, P.O. Box 1524, Kathmandu 44600, Nepal; prakashsunder@hotmail.com; 2 Centre for International Health, University of Bergen, P.O. Box 7800, 5020 Bergen, Norway; ram.chandyo@uib.no (R.K.C.); tor.strand@uib.no (T.A.S.); 3 Department of Nutrition, Harvard T.H. Chan School of Public Health, 665 Huntington Avenue, Boston, MA 02115, USA; a.thorne-lyman@cgiar.org (A.L.T.-L.); mina@hsph.harvard.edu (W.W.F.); 4 World Fish, Malaysia, GPO 10670, Bayan Lepas, Penang 11960, Malaysia; 5 Oslo and Akershus University College of Applied Sciences, P.O. Box 4, St. Olavs plass, 0130 Oslo, Norway; sigrun.henjum@hioa.no; 6 Section for Pharmacology, Department of Clinical Science, University of Bergen, 5020 Bergen, Norway; per.ueland@ikb.uib.no; 7 Bevital AS, c/o Helse Bergen, Jonas Lies veg 87, 5020 Bergen, Norway; bjorn.midttun@uib.no; 8 Department of Epidemiology and Global Health and Population, Harvard T.H. Chan School of Public Health, 665 Huntington Avenue, Boston, MA 02115, USA; lag111@mail.harvard.edu; 9 Innlandet Hospital Trust, 2629 Lillehammer, Norway

**Keywords:** vitamins, infant, Nepal, methylmalonic acid, homocysteine

## Abstract

Vitamin deficiencies are known to be common among infants residing in low- and middle-income countries but relatively few studies have assessed several biochemical parameters simultaneously. The objective of the study was to describe the status of vitamins (A, D, E, B_6_, B_12_ and folate) in breastfed infants. We measured the plasma concentrations of *trans retinol*, 25 hydroxy vitamin D, α-tocopherol, pyridoxal 5′-phosphate, cobalamin, folate, methylmalonic acid, homocysteine, hemoglobin and C-reactive protein from 467 randomly selected infants. One in five (22%) was deficient in at least one vitamin. Mean (SD) plasma folate concentration was 73 (35) nmol/L, and no infant in the sample was folate deficient. Vitamin B_6_ deficiency and vitamin B_12_ deficiency was found in 22% and 17% of the infants, respectively. Elevated plasma methylmalonic acid or total homocysteine concentration was found in 82% and 62% of infants, respectively. Fifteen percent of infants were vitamin A deficient and 65% were marginally deficient in vitamin A. Fewer than 5% of infants had low plasma vitamin D concentration or vitamin E concentration (α-tocopherol <9.3 µmol/L). Our results illustrate the importance of continued supplementation campaigns and support the expansion of food fortification and dietary diversification programs that target children and women in Nepal.

## 1. Introduction

Vitamins and minerals are essential for children’s physical health, growth and cognitive development [1,2]. Worldwide more than two billion people, mostly residing in low-income countries, are deficient in key vitamins and minerals [3]. The estimated prevalence of vitamin deficiencies among Nepalese children has ranged from 6% to 59% [4]. Avoiding vitamin deficiencies is particularly important in infants as this period represents an extremely important time for growth and development and complementary foods often lack the required nutrient content and quality [5].

Vitamin A deficiency, even in subclinical form, increases the risk of childhood morbidity and mortality [6,7]. In Nepal, a recent national survey reported that only 47% of children aged 6 to 23 months consumed foods rich in vitamin A on the day prior to the interview [8]. Vitamin D is important for calcium metabolism, bone health, and immunity [9,10], and may also be important in cognitive development [11] and for preventing infections, including pneumonia [12]. Vitamin D deficiency is more common among infants who have poor sunlight exposure or whose mothers are deficient [13]. Vitamin E is required for modulating oxidative stress [14]. Folate, vitamin B_12_, and vitamin B_6_ are crucial for the methylation cycle. Deficiencies of these vitamins may affect growth and cognitive performance in children [15,16,17]. Most of these vitamins are also essential for antibody production and for the integrity of the gastrointestinal and respiratory epithelium [18]. Prevention of common micronutrient deficiencies in the first two years of age is an important priority for low income countries [19]. However, little data exists on the prevalence of various micronutrient deficiencies and how the plasma concentration of their biomarkers varies throughout infancy. The purpose of this study was to describe the status of key vitamins (A, D, E, B_6_, B_12_ and folate, and the biomarkers total homocysteine and methylmalonic acid) in a random sample of breastfed infants, from two months of age, residing in Bhaktapur, Nepal.

## 2. Subjects and Methods

### 2.1. Subjects

The study was conducted in Bhaktapur municipality located 15 km east from the capital of Nepal (Kathmandu). Bhaktapur is a semi-urban community of approximately 80,000 inhabitants who predominantly belong to the Newar ethnic group. Agriculture is an important component of the majority of livelihoods among residents of the Bhaktapur municipality. Similar to the rest of Nepal, diet in Bhaktapur consists primarily of rice and lentils, with seasonal availability of green leafy vegetables. Meat, fish and dairy products are not regularly consumed even though most of the people are not vegetarians. Most of the pregnant women (~90%) attend regular antenatal visits at different health centers in the community and receive iron (elemental iron of 60 mg) with folic acid (400 µg) usually from the first trimester, and also calcium tablets (500 mg) from the second trimester which are continued up to 45 days postpartum [20]. The Government of Nepal distributes iron and folic acid free of cost but most of the pregnant mothers purchase it from the local pharmacy and composition may vary from one brand to another. The Government of Nepal also distributes bi-annual vitamin A supplementation to children from six months to five years.

### 2.2. Enrollment Procedure

Mother-infant pairs were identified thorough a community based surveillance. A cluster sampling procedure was used, in which we randomly selected 66 out of 160 geographic areas (called *Toles*) in Bhaktapur municipality; from these Toles, we generated a list of all eligible mother-infant pairs and randomly approached 582 pairs whom we invited to participate in this study. They were eligible to participant if the infant was from two to 12 months of age, currently breastfed, and were not diagnosed with an acute infection or a chronic or congenital disease. Details of this process can be found elsewhere [20]. A total of 500 mother-infant pairs were enrolled in the study from January 2008 to February 2009; vitamin status was analysed only in 467 infants as the volumes of the plasma samples were not sufficient from 33 enrolled infants.

Upon selection, mothers were asked to bring their children to Siddhi Memorial Hospital for administration of the household questionnaire, physical examination, anthropometric measurement, and blood collection. All mothers gave informed consent before participating in the study. Ethical clearance was approved from the institutional review board at the Institute of Medicine, Kathmandu, Nepal and the Regional Research Ethical Committee in Norway. The length of infants were measured with locally made wooden boards that were periodically calibrated. Infant’s weight-for-age, height-for age, and weight-for-height z-scores were calculated based on WHO growth standards [21]. Perinatal characteristics, as well as birth weights, were recorded based on mother’s recall or birth records. We defined breastfeeding according to the recent WHO guidelines. Exclusive breastfeeding was defined when the infant had received only breast milk form his/her mother or a wet nurse, or expressed breast milk and no other liquids or solid with the exception of drops of syrup consisting of vitamins, mineral supplements or medicines [22]. All forms were manually checked for inconsistencies and data was double entered.

### 2.3. Sample Size Calculations

Four hundred and fifty individuals were required to detect a 25% prevalence of vitamin deficiency with an absolute precision of 5%. Assuming a 10% loss-to-follow-up, we therefore enrolled 500 children.

### 2.4. Blood Sampling and Biochemical Analysis

Approximately 3 mL of whole blood was taken from the cubital vein using polypropylene tubes with lithium heparin (Sarstedt, Germany). We measured hemoglobin (Hb) on HemoCue (HemoCue, Vedbæk, Denmark) immediately after blood collection. The HemoCue was calibrated on a regular basis. The samples were then centrifuged (760× *g*, for 10 min, room temperature) and plasma was allocated into polypropylene vials (Eppendorf, Hinz, Germany). Samples were stored at −20 °C at the field site laboratory until they were transported with an ice pack to the central laboratory in Kathmandu at the end of each day. There, samples were stored at −80 °C until transport on dry ice to Norway. Blood samples were analysed at the Bevital laboratory, Bergen, Norway. Plasma vitamin B_12_ and folate levels were determined by microbiological assay based on growth support of *Lactobacillus leichmannii* and *Lactobacillus casei*, [23,24] respectively. These assays were adapted to a microtiter plate format and carried out by a robotic workstation; the within day coefficients of variation were 5% [25]. Plasma methylmalonic acid (MMA) and total homocysteine (tHcy) were analyzed using a gas chromatography mass spectrometry based on methylchloroformate derivatization [26]. Plasma vitamin B_6_, in the form pyridoxal 5′-phosphate (PLP), and fat soluble vitamins A, D, and E, in the form of *trans retinol*, 25 hydroxyvitamin D 25(OH)D and alpha-tocopherol, respectively, were measured by liquid chromatography-tandem mass spectrometry [27,28]. Throughout the paper, we use 25 (OH) D to describe the sum of 25 (OH) D_2_ and 25 (OH) D_3_, and plasma vitamin A for all-*trans retinol* values. Plasma C-reactive protein (CRP) was analysed by turbidimetric immunoassay (Tina-Quant, Roche, Germany) on Hitachi 917 (Tokyo) at the laboratory of clinical biochemistry, Haukeland University hospital in Bergen, Norway. Definitions and cut-off points for vitamin deficiencies, MMA, tHcy in plasma, and anemia are shown in Table 1.

### 2.5. Data Analysis

The statistical analyses were performed using Stata, version 13 (Stata Corp. 2013. *Stata Statistical Software:* College Station, TX: Stata Corp LP) adjusted for clustering sample design using the SVY commands. We present mean, median, and interquartile range (IQR) of vitamins A, B_6_, B_12_, folate, D, E, and metabolites tHcy and MMA. The prevalence of vitamin deficiencies was presented with 95% confidence intervals. We evaluated differences in the levels of vitamin deficiencies across ages; a *p* value of <0.05 was considered to reflect a statistically significant difference. Spearman correlation analysis was undertaken to investigate the association with different vitamins and age. As the concentrations of vitamin biomarkers might be influenced based on presence of clinical or subclinical infection, we also present association of CRP with the plasma vitamin concentrations [37].

## 3. Results

### 3.1. Baseline

Socio-demographic and anthropometric parameters of enrolled infants are shown in Table 2. The study achieved almost equal enrolment of male and female infants. The mean age (SD) of infants was 6.8 (2.9) months; 45% children were six months or younger and 9% children were born at home. Mean birth weight was 2891 g; 13% of infants were classified as low birth weight (<2500 g). More than half of the mothers (53%) were illiterate or had only up to grade 5 level education. Most of the mothers (90%) received iron supplementation during the last pregnancy and most of the iron capsules also contain folic acid. While initiation and continuation of breastfeeding is universal in this community, only half of the mothers practiced exclusive breastfeeding of their infants up to three months. Complementary feeding in the form of home-made cereal based porridge (lito) or animal or powder milk are commonly introduced at early as from one month of life due to common belief that mother milk alone is not sufficient for their children [38]. Nearly two thirds of infants below six months of age were introduced to home made cereals (lito) as complementary food whereas 10% and 7% introduced cerelac (commercially available fortified porridge) and Lactogen, respectively. Approximately 60% of infants were anaemic (Hb < 110 g/L) at enrollment; after adjusting the Hb cut-off for altitude, the proportion increased to 70%.

### 3.2. Plasma Concentration of Vitamins and Markers for B-Vitamin Status (MMA and tHcy)

The mean (SD), median, and percentile distribution, as well as status and deficiencies of vitamins and metabolites, are summarized in Table 3 and Table 4. The median (IQR) of plasma vitamin A, D, E, B_6_, B_12_ and folate were: 0.95 (0.79–1.1) µmol/L, 80 (67–93) nmol/L, 20.3 (17.6–23.6) µmol/L, 31 (20.8–47.4) nmol/L, 232 (165–321) pmol/L, 66.9 (45.3–96.8) nmol, respectively. Vitamin A deficiency (plasma *trans retinol* concentration <0.70 µmol/L) was found in 15% (95% CI: 11–18) of infants while 65% (95% CI: 61–69) were classified as marginally deficient (<1.05 µmol/L). Corresponding figures among two to six months of age were 18% and 77%, respectively.

Very few infants (<4%) were deficient in either vitamin D or vitamin E. Approximately one in five infants (22%, 95% CI: 18, 25) were vitamin B_6_ deficient; and among infants without elevated CRP concentrations the prevalence was 19%. Vitamin B_12_ deficiency was found in 17% of the infants (95% CI: 14, 20), whereas 40% (95% CI: 35, 44) were considered to have marginal B_12_ deficiency (<200 pmol/L). While none of the infants had poor folate status (<10 nmol/L), the metabolic markers MMA and tHcy were elevated in 82% and 64% of infants, respectively. Among the infants with poor vitamin B_12_ status, 93% had elevated MMA and 83% had elevated tHcy.

In addition to evaluating the prevalence of a single vitamin deficiency, we also assessed multiple vitamin deficiencies in this population. Infants who had marginal vitamin A deficiency also had 26.9%, 24.7% and 31.7% marginal deficiencies of vitamins D, E, and B_12,_ respectively. An overlap of marginal vitamin B_12_ and vitamin B_6_ deficiency was seen in 10.7%, while 18.6% of infants were marginally deficient in both vitamin B_12_ and vitamin D. Anemic infants (Hb <110 g/L) were more likely to be deficient, or marginally deficient, in at least one vitamin. Notably, 40% of anemic infants had marginal vitamin A deficiency, 25% had marginal vitamin D deficiency, and 15% and 11% were vitamin B_6_ and B_12_ deficient, respectively (Table 5).

### 3.3. Age Related Changes of Plasma Vitamins and Metabolic Markers

**Figure 1:**
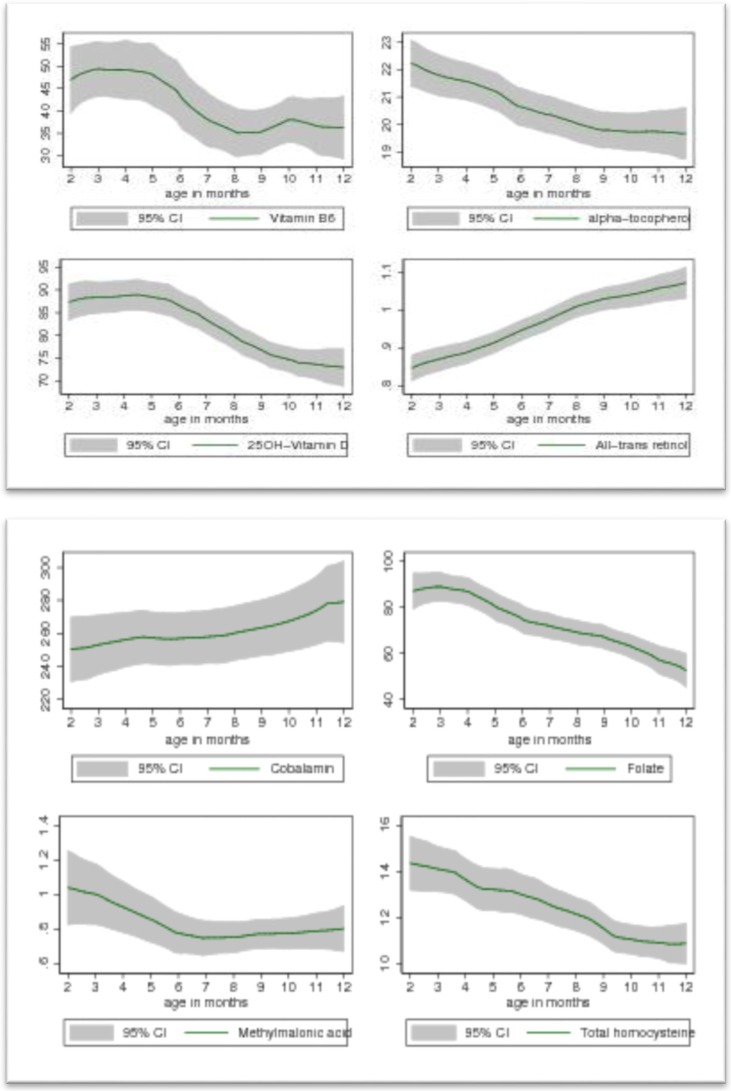
Association between vitamins of B_6_(pyridoxal 5′ phosphate nmol/L), E(α-tocopherol, µmol/L), D(25(OH)D, nmol/L), A(*trans retinol*, µmol/L) , B_12_(cobalamin, pmo/L), Folate(plasma folate, nmol/L), MMA(µmol/L) and tHcy(µmol/L) with age among 467 breastfed Nepalese infants. The Y axis indicate the mean concentration of plasma vitamins or metabolic markers. X axis indicate the age of the child. The shaded area represents 95% CI of the plasma vitamins and metabolic markers.

The concentrations of plasma vitamins and metabolic markers by age are shown in Figure 1.

Plasma vitamin A and vitamin B_12_ concentration were low at two to three months and increased throughout infancy. Plasma 25 (OH) D, α tocopherol, and folate were higher among two to three month olds and declined with age. The mean plasma vitamin A was 0.85 µmol/L among infants less than six months of age compared to a mean plasma vitamin A of 1.0 µmol/L among infants six months and older (*p* < 0.0001). Plasma PLP declined with increasing age, and was 10 nmol/L lower in infants ≥ six months of life than in younger infants (*p* < 0.003). In relation to age, tHcy and MMA were highest in younger infants (the first 6 months of life).

### 3.4. Correlation Analyses

The Spearman correlations between vitamins, metabolic markers (MMA and tHcy), and CRP are shown in Table 6. The concentrations of plasma vitamin A, α-tocopherol, PLP, and cobalamin were positively correlated with each other. However folate was negatively associated with vitamin A and cobalamin but positively associated with vitamin D. While the concentration of tHcy was inversely associated with cobalamin and vitamin A, it was positively correlated with folate. MMA was associated negatively with cobalamin and positively with folate. CRP was inversely associated with vitamin A and PLP whereas vitamin B12 was positively associated with CRP. Vitamin A, α- tocopherol, PLP and Vitamin B12 were positively associated with Hb level. All indicators of vitamins except vitamin A and cobalamin were negatively associated with the age of the child.

## 4. Discussion

Optimal vitamin status during infancy is important for many aspects of child health, growth, and development. We found that the prevalence of vitamin (A, D, E, B_6_, and B_12_) and folate deficiencies in the Bhaktapur municipality of Nepal, ranged from zero to 65% using thresholds reflecting marginal deficiency for each vitamin. One of the main challenges to the interpretation of prevalence figures of biochemical parameters for young infants is the lack of age-specific cut-off values. We therefore presented the data both in terms of the proportion of infants with deficiency and marginal deficiency as well as percentiles. Despite the timing of a national bi-annual vitamin A supplementation program that targets children six months and older, the prevalence of vitamin A deficiency, and marginal vitamin A deficiency, in Bhaktapur was fairly high (15% and 65% respectively). These findings suggested that the prevalence of Vitamin A deficiency is almost twice as high compared to another study conducted among six to eight year-old children in the Southern Nepal [39]. The relatively low concentrations of plasma vitamin A in younger infants identified by this study may be due to low maternal intake of vitamin A rich foods during pregnancy and lactation [8] and sub-optimal breast milk concentrations of retinol [40]. In Nepal, cultural beliefs and food taboos restrict intake of foods (leafy vegetables and fruits) that are rich in vitamin A during pregnancy and breastfeeding [41], and low consumption of meat, fish and other animal source foods may also cause poor maternal nutritional status during pregnancy and breastfeeding. Furthermore, the relatively low coverage of vitamin A supplementation in younger children (six to 12 months) may also contribute for the low concentrations of plasma vitamin A reported in this population. While 92% of children aged 12 to 59 months received bi-annual vitamin A supplements, only 70% of children six to 12 months benefited from the national program [8,42].

The prevalence of vitamin D and vitamin E deficiencies in this study population was lower than that of vitamin A, but marginal deficiencies of these vitamins were common and were seen in more than a third of infants. Unfortunately, there is limited information available on status of vitamin D and E from infants in the South Asia. A recent study conducted in rural Nepal found that 91.5% of pre-school children were vitamin D deficient using a threshold of 25 (OH)D <50 nmol/L) [43], indicating a substantially higher burden of vitamin D deficiency in Nepal than what we report in our study. We found only five infants who were at the threshold of 25 (OH)D <40 nmol/L, a cut off value which was used to set estimated average intake value of vitamin D intake and often recommended to define vitamin D deficiency in population. The differences between our results and those findings may be explained by differences in age, study location, methods to sample and analyse vitamin D, as well as sunlight exposure. While the study from rural Nepal assessed vitamin D status among pre-school children, our study population consisted of children from two to 12 months of age. Furthermore, in Bhaktapur it is common to give regular sunbaths with oil massage to newborn infants for the first three months of life. This tradition may expose infants in Bhaktapur to direct sunlight, thereby stimulating the production of vitamin D. These factors may explain the higher 25(OH) D concentrations reported in our study.

Of the water-soluble vitamins, we found a high prevalence of vitamin B_12_ (17%) and vitamin B_6_ (22%) deficiencies, but not folate deficiency. The concentration of plasma PLP was inversely correlated with the CRP concentration. The close relationship of vitamin B_6_ with inflammation has been reported in other studies [44,45], and there is a concern that lower plasma PLP concentration does not necessarily represent vitamin B_6_ status in the presence of inflammation [46]. Together with vitamin B_12_ and folate, vitamin B_6_ plays a crucial role in homocysteine metabolism in the methylation cycle [47]. However, our results did not show higher plasma concentration of tHcy among infants with vitamin B_6_ deficiency, indicating that the majority of metabolic alteration of tHcy is related to vitamin B_12_ deficiency in this community.

The high folate concentration and positive correlation with tHcy may also be a result of the folate trap phenomenon [48]. In Nepal, the diet primarily consists of rice and lentils with seasonal access to green leafy vegetables. Few regularly consume meat, milk or fish, the main sources of B_12_ in populations lacking access to fortified foods [49]. The relatively high prevalence of marginal vitamin B_12_ deficiency (40%) among infants is consistent with the low dietary diversity observed in this population [49], and reflects the high prevalence of marginal vitamin B_12_ deficiency observed among children and women from the same community, as well as in other communities across Nepal and India [50,51,52]. While vitamin B_12_ concentration was positively correlated with age, folate concentration was inversely associated with age. This is likely explained by concentrations of folate in breast milk which is high even when mothers have a low intake of this nutrient. According to national health recommendations, pregnant mothers regularly receive folic acid and iron supplements throughout the pregnancy and lactation period [53].

Most of the infants had more than one marginal vitamin deficiency. This is consistent with other studies from Nepal that report a high burden of multiple micronutrient deficiencies among pregnant and lactating mothers and children [20,36,39]. The concentrations of vitamin A, E, B_6_ and B_12_ in our study were positively correlated. We also found a correlation between anemia and deficiencies in vitamins A, B_6_, B_12_ and folate, highlighting the potential contributions of other nutrients to the etiology of anemia among these young infants. Iron deficiency anemia is consistently reported as one of the main public health problems in Nepal [54] [20,36,39]. Depleted iron stores were found among 20% of infants only whereas 70% were anemic (Hb < 113 g/L), a detailed description of other iron parameters have been published elsewhere [55]. Poor intake of iron rich complementary foods and physiological anemia at three to seven months of age are probably underlying causes of anemia in this population. Young infants are entirely dependent on breast milk which is low in iron concentration [56], and local complementary foods are usually not fortified with iron [38].

Although they are only needed in tiny amount, micronutrient malnutrition is a public health problem in developing countries. Most infant diets in the community contained insufficient amounts of vitamins and minerals because of poor food diversity. To prevent deficiencies of micronutrients, food fortification should be expanded from current pilot projects in some parts of Nepal, and further efforts to diversify the food supply are needed [57,58].

## 5. Conclusions

In this peri-urban population in the Kathmandu Valley, we observed a high prevalence of marginal deficiency of vitamins among breastfed infants. The levels of vitamin A deficiency documented among infants in this study highlight the importance of continued vitamin A supplementation campaigns, particularly for infants six to 12 months of age who have lower coverage rates than children aged 12 to 59 months through the national vitamin A program. Our findings also illustrate the importance of expanding food fortification and dietary diversification programs targeting children and women.

## Figures and Tables

**Table 1:** Definition and cut-off values of anemia and plasma concentration of vitamin and metabolites among 467 infants in Bhaktapur, Nepal.

Definition	Indicators	Cut off
Anemia [29]	Hemoglobin (Hb)	<110 g/L
Altitude adjusted anemia [30]	Hb	<113g/L
Vitamin B12 deficiency [31]	Cobalamin	<150 pmol/L
Marginal vitamin B12 deficiency	Cobalamin	<200 pmol/L
Folate deficiency [31]	plasma folate	<10 nmol/L
Vitamin B6 deficiency [32,33]	pyridoxal 5′-phosphate	<20 nmol/L
Vitamin A deficiency [33,34]	*trans retinol*	<0.70 µmol/L
Marginal vitamin A deficiency [34]	*trans retinol*	<1.05 µmol/L
Vitamin D deficiency [35]	25 hydroxyvitamin D (25(OH)D)	<50 nmol/L
Marginal Vitamin D deficiency	25(OH)D	<75 nmol/L
Vitamin E deficiency [36]	α- tocopherol	<9.3 µmol/L
Marginal vitamin E deficiency	α- tocopherol	<18.9 µmol/L
Hyperhomocysteinemia [17]	Total homocysteine (tHcy)	>10 µmol/L
Hypermethylmalonic acidemia	Methylmalonic acid (MMA)	>0.28 µmol/L

**Table 2:** Baseline characteristics among 467 healthy infants participating in micronutrient survey in Bhaktapur Nepal.

Characteristics	%	Mean	SD
Age in months		6.8	2.9
Age ≤6 months	45		
First born child	42		
Male child	56		
Home delivery	9		
Birth weight, gm *		2891	492
Low birth weight (<2500 gm)	13		
**Demographic features:**			
Illiterate or up to grade 5 education of mother	53		
Illiterate or up to grade 5 education of father	5		
Not working mother	65		
Mother age		25.5	2.2
Family residing in rented house	37		
Family staying in joint family	51		
Family having own land	54		
**Breastfeeding status:**			
Exclusive breastfeeding for 3 months or more **†**	51		
Exclusive breastfeeding for 6 months or more	17		
**Nutritional status:**			
Underweight (weight for age Z score ≤ 2)	5		
Stunting (length for age Z score ≤ 2)	9		
Wasting (weight for length Z score ≤ 2)	2		
Hemoglobin, g/L		107	12
Anemia (hemoglobin <110 g/L)	59		
Anemia (hemoglobin <113 g/L)	70		
**Infection/inflammation:**			
C-reactive protein >5 mg/L **‡**	20		

* Among 438 newborns from whom birth weights were recorded; **†** exclusive breastfeeding defined as child only consuming breastmilk and no other fluid or food except medicine, prevalence calculated for those who had passed the relevant age; **‡** among 463 infants.

**Table 3:** Mean and percentile distributions of micronutrient and total homocysteine and methylmalonic concentrations among infants in Bhaktapur, Nepal.

Mean/Centile	Vit A (µmol/L)	Vit D (nmol/L)	Vit E (µmol/L)	Vit B_6_ (nmol/L)	Vit B_12_ (pmol/L)	Folate (nmol/L)	tHcy (µmol/L)	MMA (µmol/L)
Mean (SD)	0.96 (0.25)	82 (21)	20.6 (4.9)	41.3 (35.8)	260 (131)	73 (35.0)	12.5 (5.5)	0.84 (0.88)
5%	0.58	52	12.3	13.7	104	24.7	6.4	0.19
25%	0.79	67	17.6	20.8	165	45.3	8.6	0.33
50%	0.95	80	20.3	31	232	66.9	11.2	0.55
75%	1.1	93	23.6	47.4	321	96.8	14.6	1
95%	1.3	145	27.4	74	442	129.7	19.4	1.8

Vit A = *trans retinol*, Vit D = 25(OH)D, Vit E = (α-tocopherol), Vit B_6_ = pyridoxal 5′ phosphate, Vit B_12_ = cobalamin, tHcy = total homocysteine, MMA = methylmalonic acid.

**Table 4:** Prevalence of vitamin (A, D, E, B_6_, B_12_, folate) deficiencies and metabolic markers (MMA and tHcy) in healthy infants of Bhaktapur, Nepal (*n* = 467).

Vitamins	*n*	% below or above Cut-off	95% CI
Vitamin A (*trans retinol*)	68	15	11, 18
Marginal vitamin A	304	65	61, 69
Vitamin D 25(OH)D	17	3.6	1.9, 5.4
Marginal vitamin D 25(OH)D	191	41	36, 45
Vitamin E (α-tocopherol)	6	1.3	0.3, 2
Marginal vitamin E	137	36	31, 40
Vitamin B_6_ (pyridoxal 5′ phosphate)	101	22	18, 25
Vitamin B_12_ (cobalamin)	80	17	14, 20
Marginal vitamin B_12_ (cobalamin)	185	40	35, 44
Folate	0	0	0
Hypermethylmalonic academia	384	82	78, 85
Hyperhomocysteinemia	289	62	57, 66

**Table 5:** Co-existence of multiple vitamin deficiencies and anemia among 467 breastfed infants in Bhaktapur, Nepal.

**Vitamins**	**%**								
Vitamin D	0.9	2.3							
Marginal vit D	5.3	25.9							
Vitamin E	0.6	1.3	0	0.9					
Marginal vit E	7.7	24.6	3	17.5					
Vitamin B_6_	4.3	4.3	2.3	10.2	0.4	11.3			
Vitamin B_12_	3	12.4	1.3	2.1	0	8.3	4.5		
Marginal vit B_12_	8.1	31.7	2	18.6	0.9	21.6	10.7		
Anemia	8.8	40	2.3	24.8	0.6	22.7	14.5	11.1	31.2
**Deficiencies of**	**Vit A**	**Marg vit A**	**Vit D**	**Marg vit D**	**Vit E**	**Marg vit E**	**Vit B_6_**	**Vit B_12_**	**Marg vit B_12_**

Cut off of vitamins deficiencies: vitamin A ≤ 0.70 µmol/L, marginal vitamin A ≤ 1.05 µmol/L, vitamin D ≤ 50 nmol/L , marginal vitamin D ≤ 75 nmol/L, vitamin E ≤ 9.3 µmol/L, marginal vitamin E ≤ 18.9 µmol/L, vitamin B_6_ ≤ 20 nmol/L , vitamin B_12_ ≤ 150 pmol/L, marginal vitamin B_12_ ≤ 200 pmol/L. Anemia = Hb <113 g/L.

**Table 6:** Correlations for vitamin status, MMA, tHcy, CRP, Hb and age among healthy infants of Bhaktapur Nepal (*n* = 467).

	25(OH)D	α-Tocopherol	PLP	Cobalamin	Folate	MMA	tHcy	CRP	HB	Age
***Trans retinol***	−0.03 *p = 0.42*	0.19 *p < 0.0001*	0.12 *p < 0.007*	0.10 *p = 0.023*	−0.23 *p < 0.0001*	0.05 *p = 0.2*	−0.18 *p = 0.0001*	−0.3 *p < 0.0001*	0.12 *p < 0.005*	0.36 *p < 0.0001*
	**25(OH)D**	0.17 *p =* 0.0001	0.13 *p =* 0.005	−0.05 *p =* 0.23	0.3 *p <* 0.0001	−0.02 *p =* 0.54	−0.07 *p =* 0.12	−0.02 *p =* 0.7	−0.05 *p =* 0.28	−0.28 *p <* 0.0001

		**α-Tocopherol**	0.29 *p <* 0.0001	0.22 *p <* 0.0001	0.01 *p =* 0.79	−0.08 *p =* 0.06	−0.004 *p =* 0.92	−0.05 *p =* 0.25	0.01 *p =* 0.10	−0.21 *p <* 0.0001
	
			**PLP**	0.10 *p =* 0.026	0.05 *p =* 0.23	0.03 *p =* 0.42	−0.08 *p =* 0.06	−0.21 *p < 0.0001*	0.22 *p < 0.0001*	−0.21 *p <* 0.0001
		
				**Cobalamin**	−0.25 *p <* 0.0001	−0.36 *p <* 0.0001	−0.52 *p <* 0.0001	0.11 *p =* 0.01	0.12 *p* = 0.006	0.05 *p =* 0.21
			
*NS = p-value > 0.05*					**Folate**	0.15 *p < 0.001*	0.32 *p < 0.0001*	0.03 *p = 0.41*	0.03 *p = 0.52*	−0.31 *p < 0.0001*
*MMA = Methylmalonic acid*										
*tHcy = Total homocysteine*						**MMA**	0.45 *p <* 0.001	−0.13 *p = 0.005*	−0.02 *p = 0.66*	−0.07 *p = 0.12*
*Hb = Hemoglobin*										
*PLP = Pyridoxal 5′phosphate*							**tHcy**	−0.04 *p = 0.35*	−0.13 *p < 0.001*	−0.21 *p < 0.0001*
						
								**CRP**	−0.04 *p = 0.35*	0.06 *p = 0.14*
							
									**Hb**	−0.07 *p = 0.09*
								
